# The Book World of Medicine and Science

**Published:** 1894-01-20

**Authors:** 


### The Book World of Medicine and Science.

### BOOK REVIEWS.

The Supernatural: Its Origin, Nature, and Evolution. By John H. King. (Williams and Norgate.)

It is impossible to read many pages of "The Supernatural: Its Origin, Nature, and Evolution," without recalling St. Paul's words, "The invisible things of Him from the creation of the world are clearly seen, being understood by the things that are made, even His eternal power and Godhead." The author does not appear to be able to make any distinction between superstition and faith. Indeed, all faith to his mind must be superstition, and, as a consequence, there can be nothing known about God; or, to use the words with which the book concludes, "The highest form of divinity we can ever know is human goodness." A great part of the book is taken up in showing that the supernatural, or rather the impressions which mankind has derived of the supernatural, are the result of evolution. No revelation, therefore, has ever taken place. It is easy enough to amass evidence of the prevalence of superstition in nearly every quarter of the globe. But the author seems to be quite blind to the fact that wherever Christianity has prevailed, magic, witchcraft, charms, spells, &c., have ceased to exercise the influence which they once did upon the human mind, and a rational faith has taken their place, not altogether, but quite sufficiently for those who have eyes to see to recognise a distinction in results as different as light from darkness. Let the author, for instance, only study the condition of some of the Melanesian islands, where Christianity has obtained considerable influence of late years, and compare their state with islands still in darkness, and he could hardly run the two conditions into one theory of evolution depending upon a growth in the minds of the savages.

We take grave exception to a statement in the preface as a specimen of the fallacy pervading the book, viz., "There is not a town in England, or in Europe, in which witchcraft is not extensively practised." If this is still the case we pity the towns; if in addition to their many manifest sins they are still so degraded by superstition, our modern pessimists must have the best of the argument. We hold, on the contrary, that Europe, and England especially, can show a distinct improvement since the days of barbarism coincident with the spread of the Christian faith. Superstition is still rife in places, and occupies a modified form in many minds little suspicious of its taint. But to make such a hopeless confusion between heathen darkness and rational faith for the sake of bolstering up an evolutionist hobby must inevitably lead (as it has in this case) to the denial of a Personal God. That many superstitions may have been evolved gradually in the human mind we do not deny. On the other hand, the view that superstition is the deterioration of a once better faith, the degradation of a once clearer knowledge, has much to recommend it.

"Myxœdema, and the Effects of Climate on the Disease." By A. Marius Wilson, M.D. (London: The Scientific Press, Limited, 1894.)

The subject of myxœdema and its treatment by preparations of the thyrord gland has been brought very prominently before the profession during the last two years, but previous to that the disease was often unrecognised when it occurred, and the treatment was as unsatisfactory as our knowledge of its symptoms and pathology. The need for a concise and readable account of the disease and its treatment will therefore be very widely felt, by senior practitioners especially, and it is to meet this want that the Scientific Press have published this little work by Dr. Marius Wilson, which is brief, accurate, and to the point.

### JANUARY REVIEWS.

In the Nineteenth Century Dr. Steeves brings forward a scheme for sanitary insurance, which would certainly commend itself to many householders if it could be proved practicable. Dr. Steeves believes that to ensure a healthy dwelling the drains should be tested three or four times a year by qualified persons, and to provide that the necessary steps should be taken without undue cost to the landlord or occupier, he proposes the establishment in each city or district of a sanitary protective and insurance association. In return for a yearly premium the association will investigate the sanitary condition of any building, issue certificates respecting it, provide for its being maintained in a sound sanitary state, and insure such buildings as it considered satisfactory against any subsequent defects. Dr. Steeves considers that there is only a small proportion of property being erected at present and passed by the best sanitary authorities that could be considered "a fair risk" for such insurance. So after all it is those least in need of protection who would profit. Professor Huxley contributes a very touching and appreciative notice of his friend Professor Tyndall, containing many very interesting personal details. Prince Krapotkin has one of his able articles on "Recent Science," embracing the most recent theories on the glacial period, and a review of the researches conducted by experimenters in this country into the transmission of the electrical discharge in vacuum tubes.

The Contemporary is an unusually suggestive number. Mr. Alfred Russell Wallace sketches a beautiful little plan for preserving the House of Lords from an untimely fate, and would fain persuade himself and his readers that the Lords only require to have the sweet reasonableness of this proposal brought to their notice to catch at "the honourable mode of escape which it offers from a difficult position." There is a good inquiry into the rise and development of anarchism by Karl Blind, pointing out that amid every noble and promising movement aiming at social reform a group of crazy fanatics are found in whom the merely destructive spirit develops itself. Other labour articles are by Professor Cunningham, Emerson Bainbridge, and Mr. Dunn Gardner.

The New Review opens the year well under altered conditions. The short story, which is the most decided innovation, may be considered powerful, but is undeniably objectionable. The beautiful illustrations which accompany Professor Max Muller's article on "The New Museum and the Sidon Sarcophagi" are a better novelty. The Anarchists are the subject of papers by "Z" and by Ivanoff, and Mr. Augustine Birrell has a terse declaration of opinion on "Disestablishment in England."

Dr. Thin subjects the Leprosy Commission to very searching criticism in the Fortnightly Review. He combats with special vigour the hypothesis of the Commissioners that direct contagion may be practically disregarded in India, because the disease in most cases arises *de novo*. He maintains that there is no evidence to show that the bacillus can exist outside the body except in rare and exceptional conditions, and that rigid isolation and supervision can alone succeed in bringing about a diminution of the plague.

The National Review has not much that is of special note this month. Mr. Alfred Austin concludes his charming article on "The Garden that I Love," which have brought a monthly perfume of roses and lilies among the sterner matters of debate this winter. Mr. Edmund Garrett contributes a very lively article on "The Loss of the United States of Africa," and Captain Maude has an ingenious theory to propound on "Imperial Insurance," *apropos* of the present "naval scare."

Sir Benjamin Richardson says much that is worth reading in Longmans on "The Athletic Life." He lays considerable stress on the value of endurance sustained by force of will in all athletic contests, and has much characteristic advice to give on the subject of diet and abstinence from alcohol. He defines the athletic age as lying between 18 and 38 years of age, before and after which ages he considers no very violent exertion should be attempted.

